# Different locations but common associations in subcortical hypodensities of presumed vascular origin: cross-sectional study on clinical and neurosonologic correlates

**DOI:** 10.1186/1471-2377-14-24

**Published:** 2014-02-05

**Authors:** João Sargento-Freitas, Ricardo Felix-Morais, Joana Ribeiro, Ana Gouveia, César Nunes, Cristina Duque, João Madaleno, Fernando Silva, Cristina Machado, Gustavo Cordeiro, Luís Cunha

**Affiliations:** 1 Neurosonology Laboratory, Coimbra University and Hospital Centre, Coimbra 3000-075, Portugal; 2 Neurology Department, Coimbra University and Hospital Centre, Coimbra, Portugal; 3 Neuroradiology Department, Coimbra University and Hospital Centre, Coimbra, Portugal; 4 Medicine Faculty of Coimbra University, Coimbra, Portugal; 5 Internal Medicine Department, Coimbra University and Hospital Centre, Coimbra, Portugal

**Keywords:** White matter lesions, Ischemic leukoencephalopathy, Small vessel disease, Transcranial doppler, Neurosonology, Stroke, Atherosclerosis

## Abstract

**Background:**

Subcortical hypodensities of presumed vascular etiology (SHPVO) are a clinical, radiological and neuropathological syndrome with a still largely unexplained pathophysiology. Parallel to the clinical heterogeneity, there is also recognised cerebral topographical diversity with undetermined etiological implications. Our aim is to assess clinical and neurosonological predictors of SHPVO according to their location.

**Methods:**

Cross sectional analysis of consecutive patients that underwent neurosonologic evaluation and head CT within one month, during a one year period. We excluded patients with absent temporal sonographic window, any pathology with a possible confounding effect on cerebral arterial pulsatility, atrial fibrillation and other etiologies of white matter diseases. The mean pulsatility index (PI) of both middle cerebral arteries was measured in the middle third of the M1 segment; intima media thickness was evaluated in the far wall of both common carotid arteries. SHPVO were rated by analysis of head CT in deep white matter (DWMH), periventricular white matter (PVWMH) and basal ganglia (BGH). We conducted a multivariate ordinal logistic regression model including all clinical, demographic and ultrasonographic characteristics to determine independent associations with SHPVO.

**Results:**

We included 439 patients, mean age 63.47 (SD: 14.94) years, 294 (67.0%) male. The independent predictors of SHPVO were age (OR = 1.067, 95% CI: 1.047-1.088, p < 0.001 for DWMH; OR = 1.068, 95% CI: 1.049-1.088, p < 0.001 for PVWMH; OR = 1.05, 95% CI: 1.03-1.071, p < 0.001 for BGH), hypertension (OR = 1.909, 95% CI: 1.222-2.981, p = 0.004 for DWMH; OR = 1.907, 95% CI: 1.238-2.938, p = 0.003 for PVWMH; OR = 1.775, 95% CI: 1.109-2.843, p = 0.017 for BGH) and PI (OR = 17.994, 95% CI: 6.875-47.1, p < 0.001 for DWMH; OR = 5.739, 95%CI: 2.288-14.397, p < 0.001 for PVWMH; OR = 11.844, 95% CI: 4.486-31.268, p < 0.001 for BGH) for all locations of SHPVO.

**Conclusions:**

Age, hypertension and intracranial pulsatility are the main independent predictors of SHPVO across different topographic involvement and irrespective of extracranial atherosclerotic involvement.

## Background

Subcortical hypodensities of presumed vascular etiology (SHPVO) are radiological findings defined as rounded ill-defined areas of decreased attenuation on CT with increased signal on T2-weighted MR sequences such as fluid-attenuated inversion recovery [1,2]. Their wide clinical spectrum includes memory impairment, executive dysfunction, mood disorders and gait impairment [3-6]. Recognised as a feature of small vessel disease (SVD) this clinical, cognitive, radiological and neuropathological syndrome remains with a largely unexplained pathophysiology, despite numerous scientific attempts to address the mechanisms underlying its development and progression [1,7,8]. Parallel to the clinical heterogeneity, there is also topographical diversity in these subcortical hypodensities, with involvement of deep white matter, periventricular white matter, subcortical grey matter and brainstem. Recently, an international effort was built trying to homogenize concepts, terminology and future research goals, and one of the main knowledge gaps identified was the possible differential mechanisms implied in the origin of these subcortical changes according to their location in the brain [8].

Complementing classical neuroimaging studies, neurosonological techniques offer distinctive information, namely through the possibility of evaluating cerebrovascular hemodynamics [9], conveying in vivo evaluation of the proceedings of brain response to vascular injury. Furthermore, the development of new pharmacological strategies and the clarification of new proposed mechanisms of action for older medications that have been suggested to promote a cerebral vasoactive effect, highlight potential treatment implications in elucidating the pathological impact of cerebral hemodynamics in SVD [10-14].

The primary objective of our work is to use a combined clinical, neuroimaging and neurosonologic approach to clarify possible differential mechanisms responsible for the development of SHPVO according to the site of involvement.

## Methods

### Patients

We included all consecutive patients undergoing ultrasonographic evaluation in our institution’s neurosonology laboratory during the year 2011.

**Figure 1:**
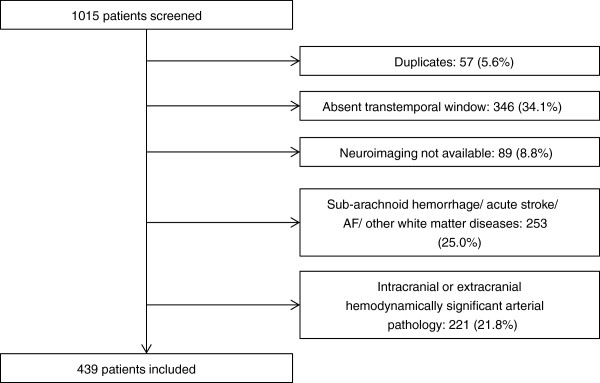
Number of patients screened, included and excluded from the study.

We excluded patients without neuroimaging exam performed within one month of neurosonologic evaluation, absent temporal sonographic window and patients with any pathology capable of a confounding effect on cerebral arterial pulsatility. Namely, we excluded all patients with stenosis >50% in any supra-aortic cervical artery [15], ≥80% stenosis in any intracranial artery [16], any grade of stenosis in a Middle Cerebral Artery (MCA) [16], occlusion in an extra or intracranial artery [15,17], all evaluations performed in context of subarachnoid haemorrhage, acute ischemic stroke, intracranial hypertension, brain death or arteriovenous malformations (Figure 1). We also excluded patients with atrial fibrillation (persistent, permanent or paroxysmal forms were considered) and specific diagnosis of cerebral white matter diseases such as Multiple Sclerosis and other inflammatory diseases. In cases of duplicate evaluations only the first study was considered. Signed informed consent was obtained for all patients. The study was approved by the local ethics committee of the Coimbra University and Hospital Centre.

### Ultrasound examination

The hemodynamic evaluation was performed through a predetermined acquisition protocol by two neurosonologists of equal experience and background using a General Electric® ultrasonography system, model Logiq 7. A linear probe of 7.5 MHz was used for the extracranial evaluation and a 3.0 MHz sector probe for the transcranial examination.

The carotid images were obtained with the patient in the supine position with the neck mildly extended and the head rotated contralaterally to the side examinated. Intima media thickness (IMT) was measured in the far wall of the distal Common Carotid Artery, 10 mm proximal to the carotid dilation, using optimal insonation angles [18,19]. The evaluation was performed in both Common Carotid Arteries and a mean value was calculated. The intracranial assessment was performed with the patient lying still in the supine position, after at least 10 minutes of rest, through an optimal transtemporal window in the middle third of the M1 segment of both MCAs. Pulsatility indexes were measured according to the Gosling formula (difference between peak systolic and lowest diastolic flow velocities, referenced to time-averaged flow velocity) [20-23]. For the analysis, the mean of pulsatility indexes (PI) from both arteries were used.

### Neuroimaging examination

All patients underwent a head CT, performed with the same scanner, a 64-slice multidectector CT (GE Lightspeed). Acquisitions were performed without contrast, from the base of the skull to the vertex. CT technical parameters included the following: matrix, 512 × 512; SFOV 25 cm; CTDlvol 76.07 mGy; 330 mAs; 120 kV; section thickness, 5 mm supratentorial, 2.5 mm infratentorial. A brain parenchymal algorithm was used to analyse the images on a computer screen. SHVPO were rated by two neuroradiologists on computer screen, blinded to clinical and ultrasound data. Ratings were done analysing CT images acquired within one month of the neurosonologic study. We graded SHPVO separately according to their location as deep white matter hypodensities (DWMH), periventricular white matter hypodensities (PVWMH) and basal ganglia hypodensities (BGH). Infratentorial lesions were not evaluated. DWMH were scored according to the age related white matter changes scale (ARWMC) as 0 (no lesions), 1 (focal hypodense areas of ≥5 mm), 2 (beginning confluence) and 3 (diffuse involvement of the entire region, with or without involvement of U fibers) [2]. BGH were also scored using ARWMC as 0 (no lesions), 1 (one focal lesion ≥5 mm), 2 (>1 lesion) and 3 (confluent lesions) [2]. For PVWMH grading we used the operacionalized ARWMC scale definitions with a score of 0 for symmetrical, well-defined periventricular hypodensities, 1 (non-symmetrical caps or bands <5 mm), 2 (periventricular hypodensities between 5 and 10 mm) and 3 (periventricular hypodensities >10 mm) [24]. In patients with territorial infarctions, these lesions were not accounted as a SHPVO.

### Clinical information

Classical vascular risk factors, namely arterial hypertension, diabetes mellitus (DM), dyslipidemia, heart failure, prior coronariopathy (both myocardial infarction and *angina pectoris* were considered), atrial fibrillation, smoking habits (active or previous), alcoholism and obesity were registered prospectively in our local neurosonology database.

### Statistical analysis

We conducted a cross sectional study of associations of clinical, demographic and neurosonologic variables with different locations of SHPVO.

Statistical analyses were performed using the SPSS 17.0 software package. Descriptive statistics of the whole population included mean (standard deviation) for continuous variables and number (percentage) for categorical variables.

Considering the large number of patients studied we conducted a multivariate analysis including all clinical, demographic and ultrasonographic characteristics to determine independent associations with SHPVO using a multivariate ordinal logistic regression model. This model was performed identically for predictors of DWMH, PVWMH and BGH. We also conducted a multivariate ordinal logistic regression model including all demographic and clinical variables but excluding the neurosonologic variables for predictors of DWMH, PVWMH and BGH.

Statistical significance for inclusion was set at p < 0.05.

## Results

During the study period 1015 patients were submitted to ultrasonographic evaluation in our cerebral hemodynamic laboratory. We excluded 576 (56.7%) patients due to exclusion criteria, thus including 439 patients (Figure 1). The descriptive analysis of clinical, demographic and neurosonologic characteristics are expressed in Table 1.

**Table 1:** Clinical demographic and neurosonologic characteristics of the whole study population

**Variable**	**Total population**
Age	63.47 (14.94)
Male gender	294 (67.0%)
Hypertension	292 (66.5%)
Diabetes mellitus	104 (23.7%)
Smoking	47 (10.7%)
Dyslipidemia	207 (47.2%)
Coronariopathy	14 (3.2%)
Heart failure	21 (4.8%)
Obesity	39 (8.9%)
Peripheral artery disease	4 (0.9%)
Alcoholism	24 (5.5%)
PI	1.0 (0.24)
IMT	0.77 (0.2)

Continuous variables are presented as mean (standard deviation), categorical variables as number (percentage). (PI = Pulsatility Index; IMT = Intima Media Thickness).

In multivariate analysis including all clinical, demographic and neurosonologic variables age, hypertension and mean PI were the only independent predictors of DWMH, PVWMH and BGH (Table 2).

**Table 2:** Results of the multivariate ordinal logistic regression with independent predictors of SHPVO

**Variable**	**Deep white matter hypodensities**			**Periventricular white matter hypodensities**			**Basal Ganglia hypodensities**		
	**OR**	**95% CI**	**p**	**OR**	**95% CI**	**p**	**OR**	**95% CI**	**p**
Age	1.067	1.047-1.088	<0.001	1.068	1.049-1.088	<0.001	1.05	1.03-1.071	<0.001
Male gender	0.874	0.569-1.345	0.541	0.714	0.471-1.082	0.112	1.298	0.825-2.042	0.26
Hypertension	1.909	1.222-2.981	0.004	1.907	1.238-2.938	0.003	1.775	1.109-2.843	0.017
Diabetes mellitus	1.158	0.741-1.81	0.52	1.283	0.826-1.992	0.268	1.2	0.764-1.885	0.429
Smoking	1.039	0.546-1.975	0.908	0.816	0.434-1.533	0.527	0.895	0.456-1.758	0.748
Dyslipidemia	1.149	0.779-1.697	0.186	1.179	0.807-1.723	0.393	1.01	0.675-1.511	0.963
Coronariopathy	0.483	0.165-1.411	0.183	0.842	0.298-2.38	0.745	0.877	0.308-2.497	0.805
Heart Failure	1.203	0.485-2.979	0.69	1.63	0.68-3.911	0.273	1.301	0.517-3.273	0.576
Obesity	1.958	0.991-3.871	0.053	1.074	0.549-2.102	0.835	1.169	0.571-2.392	0.669
Peripheral artery disease	2.346	0.345-15.961	0.384	1.783	0.267-11.909	0.551	1.366	0.195-9.584	0.754
Alcoholism	1.709	0.715-4.087	0.228	0.81	0.339-1.936	0.636	2.173	0.911-5.185	0.08
PI	17.994	6.875-47.1	<0.001	5.739	2.288-14.397	<0.001	11.844	4.486-31.268	<0.001
IMT	0.949	0.285-3.163	0.932	1.795	0.549-5.874	0.333	1.3	0.379-4.461	0.677

PI = Pulsatility Index; IMT = Intima Media Thickness; OR = Odds Ratio; 95% CI = 95% confidence interval.

In a multivariate ordinal logistic regression model including all clinical and demographic variables, but excluding mean PI and IMT, the independent predictors of SHPVO were age and hypertension for all locations. Regarding DWMH: OR = 1.087, 95%CI = 1.069-1106, p < 0.001 for age and OR = 2.023, 95%CI: 1.305-3.134, p = 0.002 for hypertension. For PVWMH, age: OR = 1.086, 95%CI: 1.068-1.104, p < 0.001 and hypertension: OR = 2.038, 95%CI = 1.329-3.125, p = 0.001. The predictors of BGH were age (OR = 1.072, 95%CI = 1.053-1.091, p < 0.001) and hypertension (OR = 1.912, 95%CI = 1.205-3.034, p = 0.006).

## Discussion

The main finding of our work is that different topographic involvement of white matter changes is independently associated with the same clinical and neurosonological features.

Small vessel disease has recently received increasing awareness to its pathogenesis. In fact, despite being recognized as one of the major worldwide causes of cognitive, physical and psychiatric impairment, the underlying mechanisms have largely maintained undetermined with paralleled treatment limitations. As such, a multinational consensus group recently published neuroimaging standards and identified the major knowledge gaps in the field. Subcortical hyperintensities of presumed vascular origin are one of the hallmark imaging presentations of SVD. Recognized to involve the deep and periventricular white matter as well as subcortical grey matter and brainstem, their etiopathogenesis has remained speculative and is indicated as one of the main controversies. In this study we tried to answer some questions still open: Are subcortical hypodensities in different locations associated with different risk factors? What is their relation with extracranial atherosclerosis and cerebrovascular resistance?

In our study the brain parenchyma was evaluated with CT. Although not ideal, CT evaluation of white matter changes is acceptable, particularly in the setting of large-scale studies [8]. Differences of MRI and CT in detecting SHPVO are primarily related to lesion size, being MRI more sensitive in detecting small SHPVO. Nonetheless, CT is as good as MRI in detecting larger, clinically relevant lesions [2] and a recent systematic review showed there is no difference between these imaging methods in diagnostic accuracy of a vascular component to dementia [25]. The ARWMC rating scale by the European Task Force on Age-Related White Matter Changes was developed with the intent to be applied in clinical trials using either CT or MRI studies, and has shown a good reliability for both imaging techniques [2]. Notably, the ARWMC scale does not include a specific grading for periventricular white matter changes. Therefore, to score this subset of SHPVO we used the operacionalized ARWMC scale [24], which has shown good inter-rater reliability and is also applicable to CT [24]. A natural limitation of CT is in the study of infratentorial lesions. As such, we chose not to evaluate subcortical changes in the brainstem. In the aforementioned consensus paper the proposed terminology for these imaging findings when involving white matter as well as subcortical grey matter and brainstem is subcortical hyperintensities of presumed vascular origin. In our study, considering we used CT as the primary imaging method, we chose to use the concept of subcortical hypodensities of presumed vascular origin (SHPVO). The choice of using CT in this study was due primarily to the possibility of including a large sample population with accurate documentation of vascular risk factors and neurosonological characterization. Moreover, the chronological proximity of both neuroimaging and neurosonology further increases the reliability of the results.

The clinical and demographic characteristics of our patients are in line with what is to be expected considering our study population and previous reports on SHPVO [4,5].

Our results show an independent association of SHPVO with older age and arterial hypertension, irrespective of the remaining risk factors. These associations remained significant even after inclusion of neurosonological variables in the regression model. The comprehension of the relationship between vascular risk factors and SVD is only at its infancy. Recent evidence supports the hypothesis that early endothelial failure, with disruption of the vessel wall architecture and leakage of plasma is a main precipitant of sporadic SVD [1]. The subsequent production of perivascular edema, which is toxic, induces rarefaction and demyelination which is seen pathologically as white matter hyperintensities on MRI or hypodensities in CT [1]. Age has invariably been associated SHPVO [5,26], with reported prevalences of up to 92% in the elderly [4,27,28]. The explanation of this common finding, might rely on the fact that cerebrovascular endothelium becomes increasingly permeable with age and some evidence suggests that age has an exponential correlation with the loss of blood-barrier function [29]. Hypertension is a key risk factor for white matter pathology but the mechanism of this association is complex and many explanations have been proposed. An highly heritable component [30,31] has been documented, and together with the fact that some patients with sustained normal blood pressure still develop SHPVO raises the hypothesis that this relation may be at the genetic locus rather than directly causal [1].

The hypothesis of early endothelial damage with blood-barrier dysfunction, favors an immune-mediated myelin damage rather than an atherosclerotic phenomenon in the etiology of SVD. This theory is reinforced by the inconsistent association of traditional vascular risk factors (other than age and hypertension) with SHPVO [27,32,33]. Our findings support this data particularly by the absent association with IMT. The quantification of the intima-media layer in the carotid artery [34] is one of the most commonly used surrogate markers of atherosclerosis and has been shown to predict future clinical atherosclerotic complications both cerebrovascular and coronary [18,35]. Our study did not show an independent association of IMT with SHPVO, once again corroborating a lack of causality of extracranial atherosclerosis with SHPVO.

Different topographic involvement of SHPVO seems to be associated with the same clinical and neurosonological features. A possible explanation for a differential topographic involvement of the same pathological condition comes from histological data reporting looser endothelial junctions in arteriolar and venular endothelium rather than capillaries [1,36]. Consequently, early endothelial dysfunction would firstly be translated in an affection of proximal perforating arterioles with lacunar infarcts in the basal ganglia, following subcortical white matter pathology from smaller capillaries in the *centrum semiovale* and periventricular area. This would favour the hypothesis of a pathological continuum from the same process with heterogeneous extension according to the primary site of physiological impairment.

**Figure 2:**
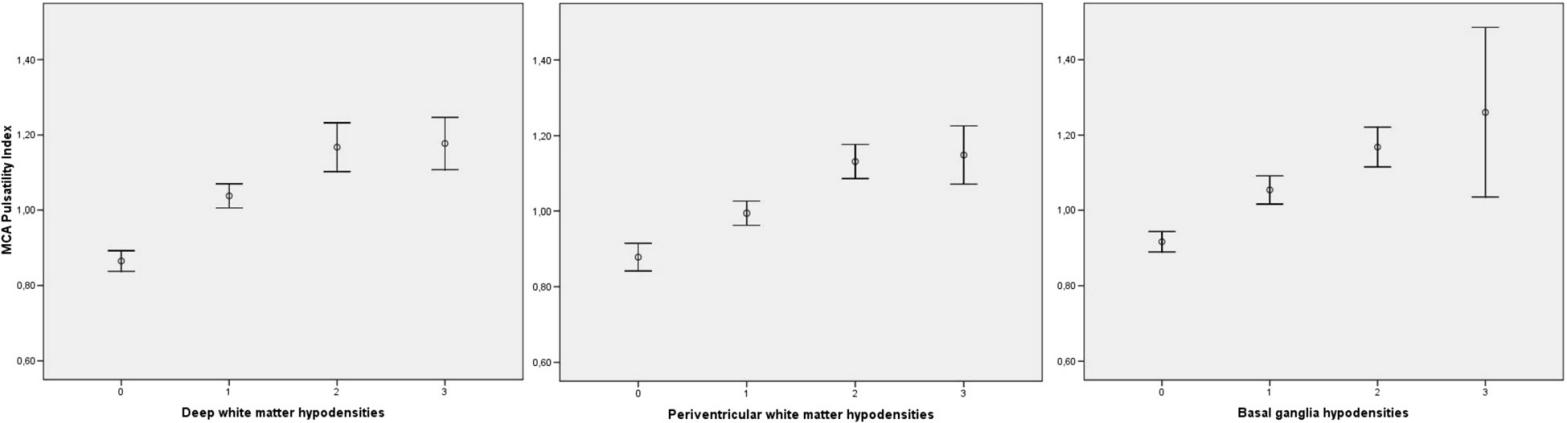
**Distribution of mean pulsatility indexes in middle cerebral arteries (MCA) according to the grade of SHPVO (0–3) for each location (deep white matter, periventricular and basal ganglia hypodensities).** Values are represented in error bars with mean and 95% confidence intervals.

Pulsatility Index of MCAs is a measure of intracranial pulsatility and is dependent on cerebrovascular resistance but also cardiac output and arterial wall pathologies with pre or post-stenotic/occlusive hemodynamic effects. As such, patient with hemodynamic confounders, as well as other pathologies capable of altering cerebral pulsatility (like subarachnoid hemorrhage, acute ischemic stroke, intracranial hypertension, brain death or arteriovenous malformations) were excluded. Patients with atrial fibrillation were also excluded due to the impossibility of correctly assessing pulsatility in an irregular pulse pattern. Furthermore, the control for cardiac failure and rest position during evaluation guaranteed that the PI observed in the MCAs was due exclusively to intracranial cerebrovascular resistance. Cerebral hemodynamics has shown an inconsistent association with SHPVO [37-40] or in aiding differentiation between types of dementia [38,39]. Our study adds evidence to this association (Figure 2) reinforcing the role of thickened arteriolar walls and thus cerebrovascular resistance in the pathogenic cascade of SHPVO, emerging as a probable consequence of endothelial dysfunction in arterioles. This can account for the positive association of PI and SHPVO, considering the early predisposition of arterioles to endothelial damage due to looser endothelial tight junctions, with leakage of plasma, perivascular edema, and at this location, disruption of the normal architecture including damaged arteriolar smooth muscle (absent in capillaries) and fibrin deposition, impairing their autoregulatory capacity. The previous discrepancies between studies are possibly explained by absent control for some situations causing altered cerebral hemodynamics and selection bias. Therefore in this study we chose to analyse consecutive patients.

These are results from a single-centred analysis, nonetheless a significant number of patients was analysed and data with clinical importance were drawn from its analysis. Moreover, blood pressure was not assessed at the time of the neurosonologic exam, which can affect arterial pulsatility. However, the effect of intracranial pulsatility was determined after adjustment for hypertension, indicating an independent association. Considering the safety and easy accessibility of transcranial ultrasound, these findings present cerebral PI as a biomarker in primary and secondary prevention of SHPVO.

The inclusion of patients with neuroimaging performed by CT alone is an obvious bias. Nonetheless, the use of validated neuroimaging scores and considering the graded analysis of SHPVO other than an absolute quantification of lesions allows an accurate interpretation of the data. Furthermore, this technique enabled the inclusion of a large number of sequential, unselected subjects, increasing the validity of the conclusions.

Multi-centre prospective trials to assess drugs potentially capable of modulating cerebral hemodynamics are mandatory and may help to finally determine the role of PI as biomarker of SHPVO. Moreover, when analysing intracranial pulsatility, confounders for hemodynamic effects should be carefully taken under consideration to minimize discrepant results.

## Conclusions

Our results indicate that age, hypertension and intracranial pulsatility are the main independent predictors of SHPVO across different topographic involvement and irrespective of atherosclerotic involvement, suggesting a possible common etiological mechanism.

## Abbreviations

BGH: Basal ganglia hypodensities; DWMH: Deep white matter hypodensities; IMT: Intima media thickness; MCA: Middle cerebral artery; PI: Pulsatility index; PVWMH: Periventricular white matter hypodensities; SHPVO: Subcortical hypodensities of presumed vascular origin; SVD: Small vessel disease; OR: Odds ratio; CI: Confidence interval.

## Competing interests

The authors declare that they have no competing interests.

## Authors’ contributions

JSF and RM wrote the final version of the article. JSF conceived the idea and analysed the data. RM, JR, AG, CN, CD, JM, and FS acquired the data. CM, GC and LC contributed with significant revision to the article. All authors read and approved the final manuscript.

## Pre-publication history

The pre-publication history for this paper can be accessed here:

http://www.biomedcentral.com/1471-2377/14/24/prepub
